# The Accuracy of ADC Measurements in Liver Is Improved by a Tailored and Computationally Efficient Local-Rigid Registration Algorithm

**DOI:** 10.1371/journal.pone.0132554

**Published:** 2015-07-23

**Authors:** Hossein Ragheb, Neil A. Thacker, Jean-Marie Guyader, Stefan Klein, Nandita M. deSouza, Alan Jackson

**Affiliations:** 1 Centre for Imaging Sciences, Faculty of Medical and Human Sciences, University of Manchester, Manchester, United Kingdom; 2 Biomedical Imaging Group Rotterdam, Departments of Medical Informatics and Radiology, Erasmus MC, University Medical Centre Rotterdam, Netherlands; 3 Cancer Research UK Imaging Centre, Institute of Cancer Research and Royal Marsden Hospital, Downs Road, Sutton, Surrey, United Kingdom; 4 The Wolfson Molecular Imaging Centre, Faculty of Medical and Human Sciences, University of Manchester, Manchester, United Kingdom; Institute of Automation, Chinese Academy of Sciences, CHINA

## Abstract

This study describes post-processing methodologies to reduce the effects of physiological motion in measurements of apparent diffusion coefficient (ADC) in the liver. The aims of the study are to improve the accuracy of ADC measurements in liver disease to support quantitative clinical characterisation and reduce the number of patients required for sequential studies of disease progression and therapeutic effects. Two motion correction methods are compared, one based on non-rigid registration (NRA) using freely available open source algorithms and the other a local-rigid registration (LRA) specifically designed for use with diffusion weighted magnetic resonance (DW-MR) data. Performance of these methods is evaluated using metrics computed from regional ADC histograms on abdominal image slices from healthy volunteers. While the non-rigid registration method has the advantages of being applicable on the whole volume and in a fully automatic fashion, the local-rigid registration method is faster while maintaining the integrity of the biological structures essential for analysis of tissue heterogeneity. Our findings also indicate that the averaging commonly applied to DW-MR images as part of the acquisition protocol should be avoided if possible.

## Introduction

The use of imaging data for decision-making, in clinical management, drug development and clinical trials is increasingly dependent on the provision of reliable, reproducible, quantitative metrics with appropriate physiological relevance [1]. Diffusion weighted magnetic resonance (DW-MR) imaging has the potential to provide such imaging biomarkers in a number of application areas, providing biomarkers with sensitivity to cell density, cellular organisation, cellular proliferation and cell death as well as its widely recognised applications in cerebral white matter mapping [2, 3]. Sensitising the MR acquisition to the free movement of water protons, at a scale equivalent to Brownian motion, as seen in the extra-vascular extra-cellular space (EES), provides data containing information about the size and configuration of the EES. This in turn can be used to imply information concerning changes in cell size or cell distribution providing an important tool for the diagnosis of cancer and for monitoring the effects of therapeutic interventions [4, 5].

DW-MR imaging relies on the acquisition of multiple matched images following application of strong magnetic gradients which de-phase and subsequently re-phase protons in a spatially dependent manner, so that small movements of protons produce signal loss. Acquisition of DW-MR data with varying strengths of gradient can be used to calculate a number of surrogate diffusional biomarkers. In cancer applications the most commonly used of these is the apparent diffusional coefficient (ADC). Any physiological movement during the acquisition phase, causing blurring or significant mis-registration between images, that are then assumed to be spatially matched, will introduce error into ADC estimates. Although studies requiring single pixel measurements of ADC are possible in static tissue, such as brain tumours [6, 7], similar approaches in organs affected by physiological motion are far more difficult [3]. This limitation is becoming increasingly problematic as recent studies highlight the importance of tissue heterogeneity within tumours [8] and the increased information content of DW-MR data that justifies analysis of ADC distribution [7, 9–11].

Previous studies indicates that in typical oncological applications, such as trials of targeted agents, there is a need to reliably detect changes in ADC of the order of 10% [3, 4]. Simple calculation show that, in order to reliably detect a 10% change in ADC in individual subjects, reproducibility must be in the order of 2% to 3% (for a reliable 2.5 standard deviation difference). In a recent multi-site reproducibility study performed in normal liver tissue [12], we found the reproducibility of ADC measurements in individuals to be in the order of 6%. This represents a best case scenario since normal tissue is homogeneous and ADC estimates are relatively unaffected by motion. ADC reproducibility in liver tumours can be greater than 10% in up to 20% of subjects largely as a result of respiratory motion which generates image blurring [13].

A number of approaches have been described to minimise the effects of respiratory motion including: breath hold acquisition strategies; respiratory triggering / gating and the use of navigator echo acquisition techniques [14–19]. Each of these is associated with significant problems and none provides a viable approach to removing errors due to motion. An alternative approach is to reduce the effects of physiological movement by image registration following acquisition [20–26]. Whole volume non-rigid alignment (NRA) has been applied in the context of ADC studies in the abdomen and breast with significant improvements in the quality of ADC images and the reproducibility of ADC measurements [20–22]. Unfortunately this approach is computationally expensive and may be subject to errors introduced by variations in tissue intensity outside the region of interest such as gallbladder emptying. In practice co-registration of a relatively small region in vicinity to the ROI is all that is required for analysis so that a simpler local-rigid alignment may suffice. This would significantly reduce execution time by avoiding the need to interpolate data at off-lattice locations.

In this paper, we examine the performance of local-rigid alignment (LRA) of DW-MR data. We have implemented a novel LRA algorithm designed specifically for use with DW-MR data and compared this with volume image registration and with on-scanner averaging of DW-MR images without alignment. In the absence of ground-truth values, we have estimated the apparent reduction in the error of ADC estimation by computing summary distribution variables in regions of real data and express the improvement as the equivalent noise that has been removed from individual ADC estimates (see [27] for the corresponding abstract).

## Materials and Methods

### Volunteer Data Acquisition

**Fig 1:**
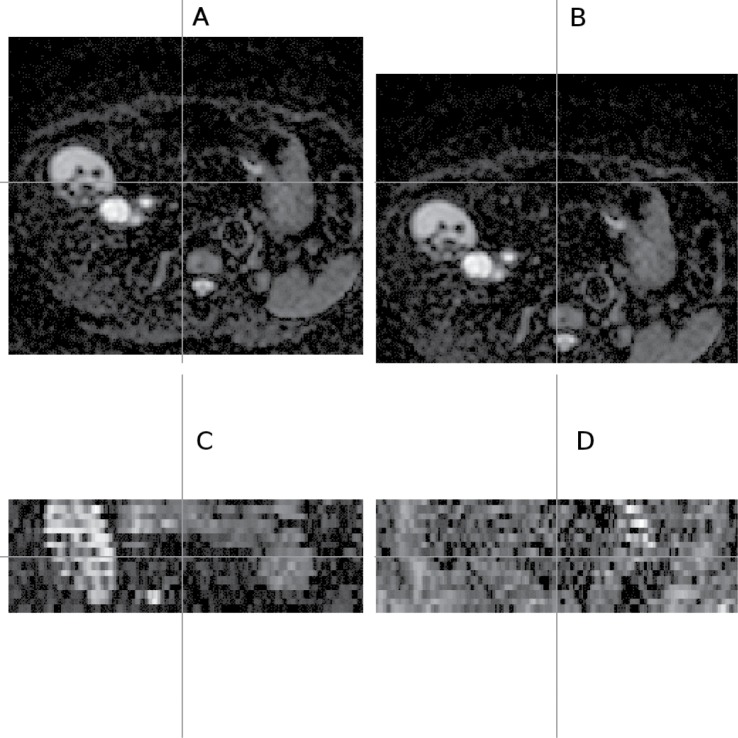
Odd-even effect observed in DW-MR data. Healthy liver DW-MR image (v3-0528) showing the odd-even effect (in the coronal view) as a result of an interleaving acquisition protocol; the coronal view images (C and D) show what is seen from the cross section of the horizontal line drawn on the transverse view slice (A and B); the two columns show different cross sections on the same volume; image volume selected is one of the repeat data-sets (out of 4) with diffusion gradient in −*x* direction; b-value = 100*s*/*mm* ^2^; gamma adjusted.

**Fig 2:**
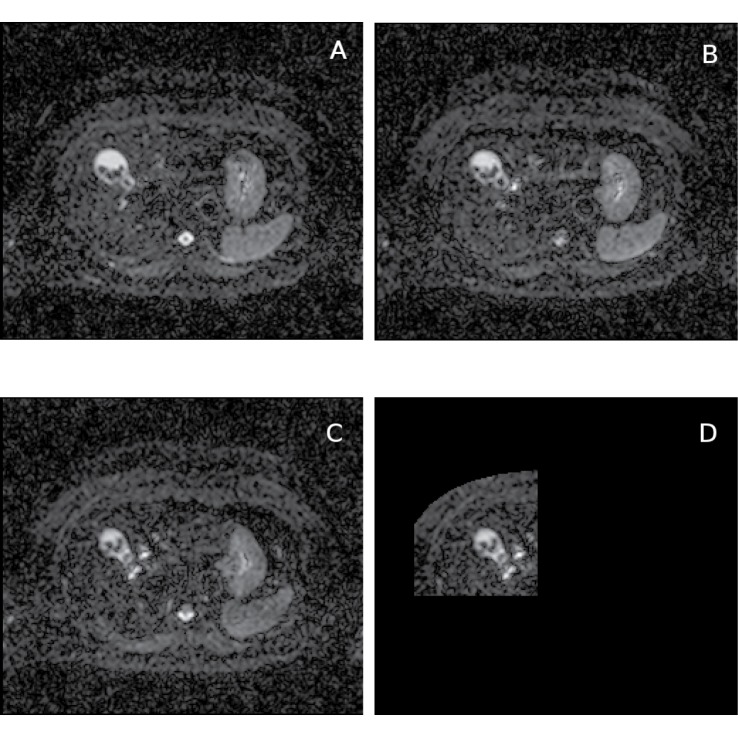
Diffusion gradient direction components of DW-MR data and the reference image. Healthy liver DW-MR image data (v3-0528-slc24); b-value = 100*s*/*mm* ^2^; selected image slice from one of the repeat data-sets (out of 4) and diffusion gradients in *y* (A) and *z* (B) directions; diffusion gradient in −*x* direction which has been used as the reference image slice for local-rigid alignment (C) and the reference image shown in the selected ROI (D); this ROI is used both in local-rigid alignment and ADC histogram analysis; gamma adjusted.

This study was approved by the local Institutional Review Board (The Royal Marsden Committee for Clinical Research) and all volunteers gave written informed consent. DW-MR images were acquired on a Siemens Magnetom Avanto 1.5 T scanner. Five fasted healthy volunteers were scanned twice within two weeks to test reproducibility of ADC in the liver. Images were acquired during free-breathing using: three b-values 100, 500 and 900 *s*/*mm* ^2^, field of view 380 × 332 *mm*, acquired pixel resolution 3 × 3 *mm*, slice thickness 5*mm*, number of slices per volume 40, and reconstructed matrix of 256 × 224 [28]. For each b-value, data was acquired using 2 separate protocols: 1) Protocol A: a single set of 3D images with on-scanner averaging of images acquired with each of the three B gradient directions [28] and 2) Protocol B: 12 sets of 3D images (40 slices per image) consisting of 4 repeated acquisitions of each of the three B gradient directions. For protocol B, data acquisition was interleaved so that each 3D image was reconstructed from 40 slices that were not contiguously acquired. In our data, odd numbered slices were acquired first followed by even numbered slices (Fig 1). For a given b-value, simple averaging of the 4 repeats and 3 gradient directions of the acquired protocol B images is expected to generate data equivalent to protocol A (Fig 2). On average, the total time for data collection corresponding to each scan session was 5 minutes 12 seconds for protocol A and 16 minutes 32 seconds for protocol B.

### Region of Interest Delineation

2D regions of interest (ROI) were delineated in the right lobe of the liver on each scan. The antero-lateral quadrant of the liver was included since motion effects in this area are significant. The size of the ROIs used were between 3580 and 8708 voxels.

### Image Registration Methods

### Non-Rigid Alignment (NRA)

The NRA method is based on Elastix [29] a publicly available open source image registration software. The deformation model is based on B-spline transformations [30] with control points spaced every 64 *mm*. The first step corrects for motion in each image, the second brings all the DW-MR images of a given scanning session into a common image space. Global registration of 3D volumes is automatic and NRA does not require ROI selection for the purpose of image registration. The deformable transformation model takes into account possible smooth continuous non-rigid misalignments caused by patient and respiratory motion during image acquisition by intra- and inter-image registration.

Intra-image registration re-aligns the spatial shifts visible between neighbouring slices caused by respiratory motion by doubling slice thickness of odd and even slices and building separate sub volumes centred at their original positions and then registering them to one another using a group-wise scheme [31] to produce a single motion-corrected image volume. Inter-image registration registers each b-value and gradient direction to a common mid-point space using a group-wise registration. Pairwise registration is then used to determine a spatial correspondence between the mid-point spaces of the 9 pairs of b-value/diffusion gradient direction. In order to reduce the number of re-sampling steps, the 36 images generated after intra-image registration are transformed to a common image space using a transformation composing the group-wise and pair-wise registrations performed in this second step.

### Local-Rigid Alignment (LRA)

LRA aligns a single reference slice to all repeat acquisitions of that slice position. It can also be used for 3D ROIs by aligning adjacent slices separately. The reference slice is taken from the lowest b-value image because it has the best signal to noise ratio (SNR; in our case, *b* = 100*s*/*mm* ^2^). Matching is performed on tissue in the right upper quadrant of the abdomen (including the liver) using body surface contours. Limits were set from observations of up to 3 pixels movements (≈ 1*cm*) in vertical and horizontal directions on each image slice and 2 slices along the z axis. There was little or no observable rotation (less than a pixel at the edge of a ROI). We allowed 5 adjacent slices per repeat data as candidates for alignment, (central slice and 2 immediate adjacent slices from either side) making 20 candidate slices for the 4 repeat acquisitions from which 4 slices with best matching scores were selected. Matching scores were derived from the 7 × 7 = 49 possible cases of shifted slices (the central pixel and 3 immediate adjacent pixels either side). The odd and even slices with best matching scores were saved so that from 4 repeats, 8 candidate shifted slices were obtained for each gradient direction. From these, 4 slices were selected from each of the 3 gradient directions, and these 12 slices were combined to produce an average aligned slice for a specific b-value. This process was repeated for other b-values. Thus, 3 b-value slices were generated from which voxel-based ADC estimates were extracted.

#### Template matching

We used an existing matching algorithm for a reference slice against a target slice based upon more conventional statistics to optimise the cost function (see below for details of optimisation). Note that here rather than using grey-level image patches and least-square differences directly, their gradients (based on differential kernels) in the horizontal and vertical directions were used. Previous work [32, 33] in medical image analysis and machine vision has demonstrated that this reduces the dependency upon absolute scaling of the data and is more suitable for matching with MRI and CT datasets.

As we did not need to consider sub-pixel shifting, we exhaustively computed the cost function for the limited number of possible variations and found the values which give the best matching score to avoid local optima. Among our candidate patches there were sub-pixel shifted versions of the region (due to the anatomical motion) and we selected only the best matching images from those available to recover some amount of sub-pixel precision whilst avoiding interpolation smoothing.

#### Optimisation

Applying the variational principle to the problem of matching two scaled noisy image patches *I* and *J* (with *N* pixels and corresponding scale factors *α* and *β*) [34], one can define the optimisation function

$$\begin{array}{c} \chi^{2}\propto \sum_{n=1}^{N}{\left(\alpha I_{n}-\beta J_{n}\right)}^{2}\;\;\;\alpha^{2}+\beta^{2}=1 \end{array}$$ (1)

It can be shown that optimising this cost function is equivalent to optimising a term which can be interpreted as the summation of the dot products of the two-component gradient vectors originated from the reference and target image patches. Based on Eq (1), *χ* ^2^ is proportional to the sum term which is theoretically sufficient for optimisation. However, we can explicitly derive formulations and rewrite the cost function in form of detailed equations as follows. Specifically, rather than using two scale factors *α* and *β*, one may use a single scale factor *γ* = *α*/*β*. For similar patches it can be shown that $\gamma =\sqrt{B/A}$ where

$$\begin{array}{c} A=\sum_{n}^{N}\;I_{n}^{2}\;;\;B=\sum_{n}^{N}\;J_{n}^{2}\;;\;C=\sum_{n}^{N}\;I_{n}J_{n} \end{array}$$ (2)

To avoid lengthy execution times, one may expand the patch similarity measure and write

$$\begin{array}{c} \chi^{2}=\frac{\sum_{n}^{N}{\left(\gamma I_{n}-J_{n}\right)}^{2}}{\sigma^{2}\left(1+\gamma^{2}\right)} \end{array}$$ (3)

$$\begin{array}{c} =\frac{\gamma^{2}\sum_{n}^{N}I_{n}^{2}+\sum_{n}^{N}J_{n}^{2}-2\gamma \sum_{n}^{N}I_{n}J_{n}}{\sigma^{2}\left(1+\gamma^{2}\right)} \end{array}$$ (4)

$$\begin{array}{c} =\frac{\gamma^{2}A+B-2\gamma C}{\sigma^{2}\left(1+\gamma^{2}\right)}\;=\;\frac{2(B-\gamma C)}{\sigma^{2}\left(1+\gamma^{2}\right)} \end{array}$$ (5)

where *σ* is the standard deviation of image noise. When choosing the reference image patch to be *J*, it follows that *B* is the constant term while *A* is varying as the target image patch is translated. Also, *σ* has a fixed value during optimisation. Hence, minimising *χ* ^2^ in Eqs (3)–(5) is equivalent to minimising

$$\begin{array}{c} \frac{-2\gamma C}{\sigma^{2}\left(1+\gamma^{2}\right)} \end{array}$$ (6)

where *γ*/(1 + *γ* ^2^) term can be eliminated during optimisation in accordance with the general proof of convergence of the Expectation Maximisation (EM) algorithm [35], so that optimising the term *C* is equivalent to optimising the *χ* ^2^.

#### Constrained movement space

The movement space defined for the LRA method covers a 3D space with 7 × 7 × 5 = 245 possible movement combinations. We observed that b-value images with good image quality (high SNR) exhibit similar movement patterns, consistent with repeated underlying respiratory motion. This movement pattern closely approximated a line in 3D. As all datasets are acquired with the same underlying cyclic respiratory motion, we used the correlation seen in good SNR data to improve the estimation of motion in lower SNR data. For the reference b-value, the shift combinations (as 3D points) for the 12 selected image slices were used to obtain a robust estimate of the line corresponding to subject specific motion. For the non-reference b-values, we allowed a subset of the original movement space, lying close to this line, to be searched. For our data, we allow all immediate neighbouring pixels of the line to be included. Constraining movement resulted in an increase in processing speed while enforcing consistent motion between b-value image data sets.

### Fits of ADC Parameters

Signal values from the corresponding b-value image slices were fitted to an exponential curve using a first order bias correction to improve the quality of fit as the noise distribution is skewed (e.g. Rician) in clinical data [36]. ADC, is the decay parameter of the curve, referred to as *D*, through a likelihood-based parameter optimisation (with log *P*(*I*∣*D*, *S*0) being the probability of the image data given the assumed parameters).

$$\begin{array}{c} \log P\left(I|D,S0\right)=\frac{-1}{2\sigma^{2}}\sum_{b_{k}}{\left[I\left(b_{k}\right)-f\left(b_{k},D,S0\right)\right]}^{2}+Q \end{array}$$ (7)

where *Q* is a constant, *f*(*b*_*k*_, *D*, *S*0) is the theoretical value of the bias-corrected exponential function and *I*(*b*_*k*_) is the signal value from the b-value image pixel. Further, *f* is a function of b-value *b*_*k*_ and the current estimate of ADC *D* and no-diffusion signal *S*0 (at *b*_*k*_ = 0), and is computed using

$$\begin{array}{c} f^{2}\left(b_{k},D,S0\right)={S0}^{2}\exp\left(-2b_{k}D\right)+\eta \sigma^{2}\;\;\;\;\;\;\;k\in \left[1,2,3\right] \end{array}$$ (8)

where *k* ∈ [1, 2, 3] refers to the three b-values *b*_*k*_ used, e.g. 100, 500 and 900*s*/*mm* ^2^ for our data. The signal value for no diffusion *S*0 (*b* = 0) is the second parameter which is estimated. *η* is a fixed value defining the amount of bias correction applied and it may be adjusted depending on the amount of image smoothing (for our data *η* was set to the theoretical value of one). An estimate of the standard deviation of noise in the image *σ* is computed from the distribution of second derivatives (for x and y) around zero [37], in a central rectangular region on the tissue. The corresponding ADC measurement software has been implemented on the project platform and has become available to all sites within the project to use.

#### Measuring change in ADC distributions

The amount of change in the width of ADC distribution was estimated via quadrature subtraction of the two ADC standard deviation values *σ*_*D*_1__ and *σ*_*D*_2__ so that

$$\begin{array}{c} \Delta \sigma_{D}=\frac{\sigma_{D_{1}}^{2}-\sigma_{D_{2}}^{2}}{|\sigma_{D_{1}}^{2}-\sigma_{D_{2}}^{2}|}\sqrt{|\sigma_{D_{1}}^{2}-\sigma_{D_{2}}^{2}|} \end{array}$$ (9)

To compute ADC reproducibility, percentage change in mean ADC values *D*_1_ and *D*_2_ for each volunteer between the two scans were computed using

$$\begin{array}{c} \Delta D\%=200\frac{|D_{1}-D_{2}|}{\left(D_{1}+D_{2}\right)} \end{array}$$ (10)

Using error propagation, it can be shown that the accuracy on Δ*D*% is given by

$$\begin{array}{c} \frac{400}{{\left(D_{1}+D_{2}\right)}^{2}}\sqrt{D_{2}^{2}\frac{\sigma_{D_{1}}^{2}}{N_{1}}+D_{1}^{2}\frac{\sigma_{D_{2}}^{2}}{N_{2}}} \end{array}$$ (11)

where *N*_1_ and *N*_2_ are the number of voxels corresponding to the mean ADC values *D*_1_ and *D*_2_.

### Assessment of Registration Performance

The data was qualitatively assessed by evaluating scatter plots of *S*0 signal against ADC for the presence of clusters of data likely to correspond to specific biological structures. Quantitative evaluation used measurement of the standard deviation of ADC estimates. Histograms of computed ADC values were used to estimate the amount of noise removed from data on the assumption that the effects of acquisition noise, genuine biological variation and motion are independent. The observed standard deviation *σ*_*D*_ of ADC values was assumed to be describable by the quadrature addition of such effects. Adjusting one of these (by for example aligning the data) and measuring a new *σ*_*D*_ while taking steps to control the other factors allows estimation of the change in this contribution (Eq 9). For an equivalent region of data the biological variation is fixed. Random noise can be controlled by the application of image blurring at a level to achieve equivalent levels of image smoothness (high spatial frequency image noise). To reach a similar degree of blurring as in NRA, the DW-MR aligned images generated using LRA were smoothed via Gaussian blurring (LRA-b, standard deviation 1 pixel). This allows the use of Δ*σ*_*D*_ as a surrogate indicator of the differences in the effects of motion related noise between images.

### Comparison of Methods/Protocols

It must be appreciated that the use of NRA techniques involves image interpolation steps which, in turn, lead to an innate blurring of the final processed image. Direct comparison of the distribution and reproducibility of calculated ADC metrics will be directly affected by this, leading to apparent improvements in reproducibility estimates (Eq 10) at the expense of image detail. This is the rationale for the generation of the blurred versions of the LRA (LRA-b) and AVG (AVG-b) data below which allow direct comparison with NRA results.

Comparison was performed using the six resulting datasets: 1) Protocol A data with on scanner averaging (PA); 2) Protocol B data averaged on repeat acquisitions and gradient directions (AVG); 3) Protocol B data corrected with LRA (LRA); 4) Protocol B data corrected with NRA (NRA); 5) Protocol B data corrected with LRA and blurred (LRA-b) and 6) Protocol B data averaged on repeat acquisitions and gradient directions (AVG) and blurred (AVG-b). 2D ROI’s selected from individual volunteer scans were used for template matching using LRA and for generating voxel-based ADC histograms. Protocol A data were compared with each of the 5 post-processed data sets.

## Results

**Fig 3:**
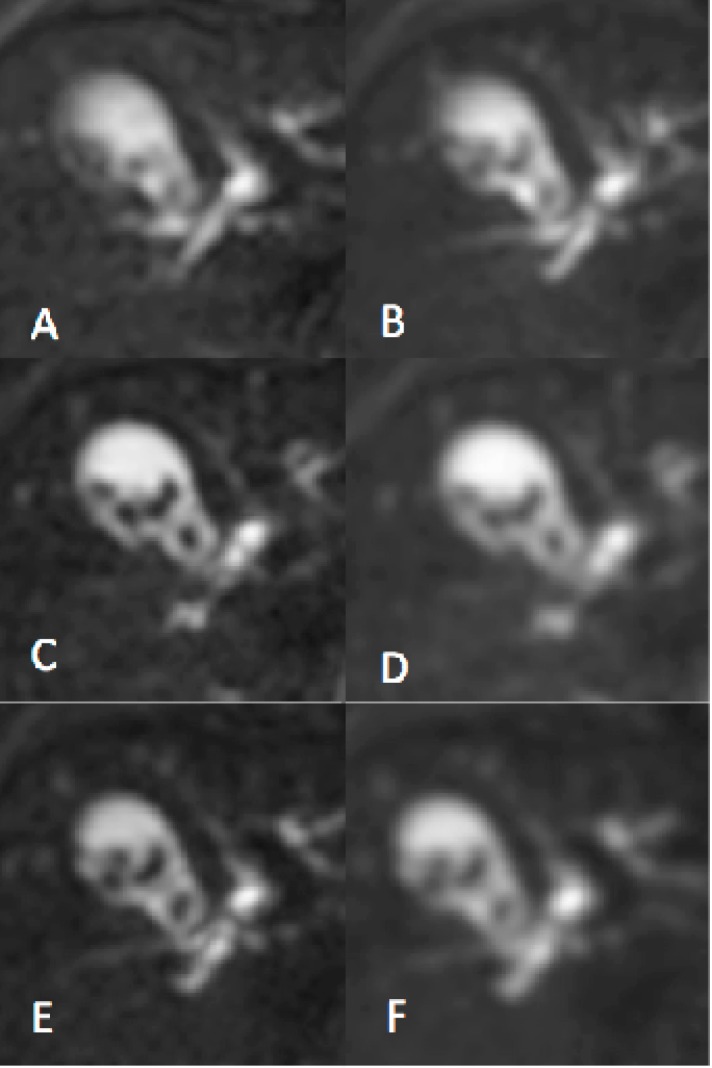
Average image slices with and without motion correction. DW-MR image data showing the gallbladder containing gallstones illustrating the differences in image sharpening with different methods (v3-0528-slc24); selected average image slices (b-value = 100*s*/*mm* ^2^); protocol A (A) and NRA method (B); simple averaging AVG (C) and the corresponding blurred version AVG-b (D); LRA method (E) and the corresponding blurred version LRA-b (F); for blurred images one pixel Gaussian blurring were applied; gamma adjusted.

Application of LRA resulted in clear improvements in subjective image quality (Fig 3E) compared to PA (Fig 3A). Image quality was also improved by simple averaging (Fig 3C). However, the improvement in image quality with NRA was compromised by the image blurring inherent in the process (Fig 3B) so that images were directly visually comparable with LRA-b and AVG-b (Fig 3F and 3D respectively). The width of the ADC histogram *σ*_*D*_ for each dataset in each subject is shown in Table 1. Table 2 shows the difference in the width of the ADC histogram Δ*σ*_*D*_ between results obtained using AVG and each of the four alternative motion correction approaches. Application of LRA and NRA resulted in reductions in both parameters compared to PA data. Comparison of LRA and NRA shows that LRA results in a larger reduction in *σ*_*D*_.

**Table 1:** Width of ADC histogram *σ*_*D*_ for a selected single slice (and region of interest) from each data-set; methods include protocol A and the corresponding average slice using protocol B with and without motion correction; same units as ADC (10^−5^ *mm* ^2^/*s*).

data-set	PA	AVG	LRA	NRA	LRA-b	AVG-b
v1-0503	27.25	30.62	27.54	22.36	23.78	26.58
v1-0515	31.05	28.85	23.58	20.53	19.31	24.69
v2-0521	54.25	55.09	44.01	63.29	42.00	53.69
v2-0528	62.04	56.12	48.15	61.49	45.62	54.24
v3-0524	56.73	67.85	65.79	67.59	62.94	64.72
v3-0528	71.82	70.71	58.64	64.25	57.18	68.55
v4-0611	32.07	32.92	30.54	25.99	26.79	28.15
v4-0614	38.30	33.94	33.85	28.27	29.53	29.61
v5-0731	32.40	32.68	33.60	29.41	29.81	28.88
v5-0809	32.59	31.06	34.34	28.94	30.09	27.01

**Table 2:** Difference in the width of ADC histogram Δ*σ*_*D*_ between results obtained using AVG and those obtained using each of the alternative methods on protocol B data (using Table 1); same units as ADC (10^−5^ *mm* ^2^/*s*); the bottom row gives the mean of Δ*σ*_*D*_ values for each method and its corresponding accuracy.

data-set	LRA	NRA	LRA-b	AVG-b
v1-0503	13.38	20.91	19.28	15.20
v1-0515	16.62	20.26	21.43	14.92
v2-0521	33.13	-31.15	35.64	12.34
v2-0528	28.82	-25.13	32.68	14.40
v3-0524	16.59	5.93	25.34	20.37
v3-0528	39.51	29.52	41.59	17.34
v4-0611	12.28	20.20	19.13	17.06
v4-0614	2.47	18.78	16.73	16.58
v5-0731	-7.80	14.24	13.39	15.29
v5-0809	-14.64	11.27	7.70	15.33
mean±accuracy	14.03±4.17	8.48±4.92	23.29±2.55	15.88±0.51

**Fig 4:**
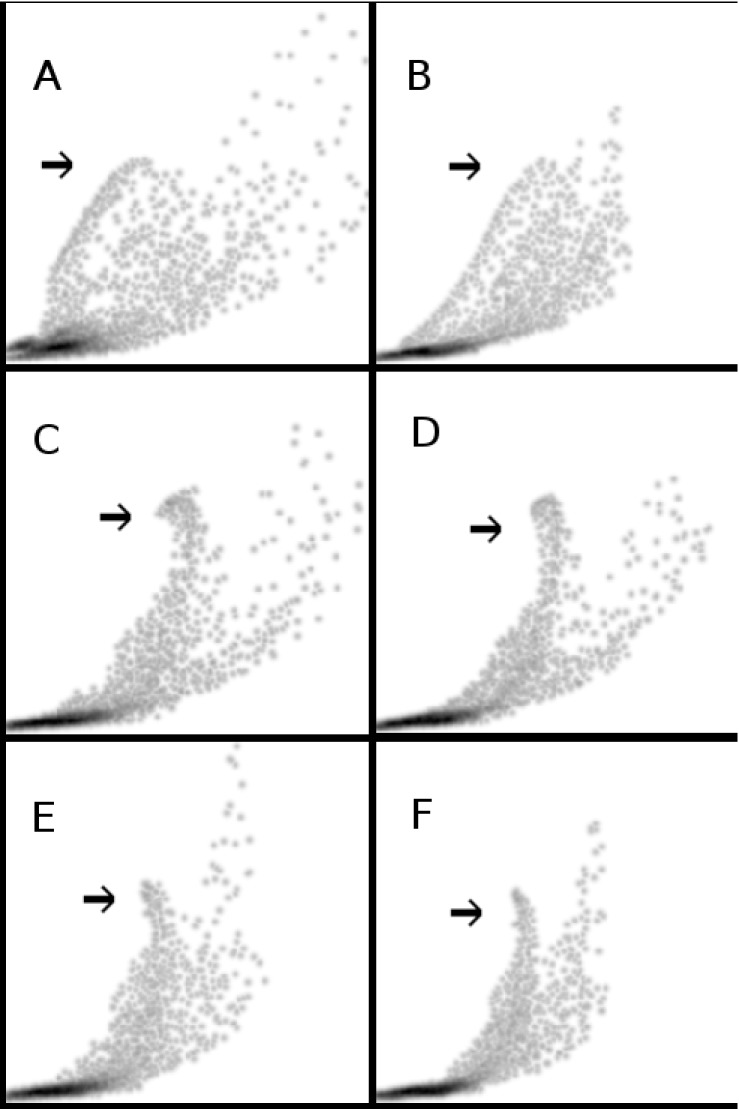
Scatter plots of *S*0 against ADC with and without motion correction. Healthy liver DW-MR image data (v3-0528-slc24); scatter plot of *S*0 signal against ADC for the identical selected 2D ROI (shown in Fig 2D); a pointer in each plot highlights an example cluster which corresponds to the bright region representing the gallbladder (as shown in Fig 3) for the study of qualitative clustering of pixels; protocol A (A) and NRA method (B); simple averaging (C) and the corresponding blurred version (D); LRA method (E) and the corresponding blurred version (F).

Although the main conclusions of the study are made based on our quantitative analysis, in Fig 4 we show the scatter plots of *S*0 signal against ADC to qualitatively assess preservation of spatial information. These plots demonstrate clusters of points where similar values of *S*0 signal and ADC are expected to correspond to a specific organ, tissue type, boundary, etc. A pointer in each plot highlights an example cluster which corresponds to the bright region representing the gallbladder (as shown in Fig 3) exhibiting high *S*0 and ADC estimates as expected. Protocol A data (Fig 4A) showed poor definition of qualitative clustering, which was improved by motion correction techniques using protocol B data. LRA-b and LRA showed the clearest improvement in qualitative clustering (Fig 4F and 4E) followed by AVG-b and AVG (Fig 4D and 4C). In contrast, the improvement in qualitative clustering following NRA was poor (Fig 4B). Visual inspection of these plots suggests slight improvements in qualitative clustering using LRA-b and AVG-b (Fig 4F and 4D) compared to LRA and AVG (Fig 4E and 4C). However, blurring does not constitute an improvement in information content, and this is why the degree of image smoothness needs to be controlled when assessing quantitative improvements in measurements. Hence, the blurred versions of the methods are included for evaluation only and are not recommended as preferred methods. These findings are in keeping with the subjective assessments of image quality described above (for Fig 3), i.e. LRA produces the most consistent improvements in ADC estimations compared to AVG, NRA and PA.

Following application of motion correction techniques, errors on estimated ADC values were dramatically reduced. Table 3 shows the percentage change in mean ADC values Δ*D*% for each volunteer between the repeat scans, calculated using measurements given in Table 4. The two bottom rows show the mean reproducibility and standard deviation (within population variability) for each method. Although NRA gave the best reproducibility (followed by LRA and its blurred version), LRA gave the lowest variability of the reproducibility metric, meaning that reproducibility was most consistent with this method across volunteers.

**Table 3:** Percentage change in mean ADC Δ*D*% from the two mean ADC values for each volunteer (using Table 4); methods include protocol A and the corresponding average slice using protocol B with and without motion correction; the two bottom rows give the mean reproducibility and standard deviation (within population variability) for each method.

data-set	PA	AVG	LRA	NRA	LRA-b	AVG-b
v1-0503-0515	11.39	7.58	2.65	0.33	1.95	7.80
v2-0521-0528	8.28	5.24	3.12	3.24	2.64	5.36
v3-0524-0528	9.47	3.08	3.84	3.53	3.78	2.25
v4-0611-0614	0.36	2.31	2.86	1.69	2.25	3.28
v5-0731-0809	1.07	1.47	3.88	0.57	4.99	1.21
mean	6.11	3.93	3.27	1.87	3.12	3.98
std dev	5.64	2.76	0.62	1.64	1.39	2.92

**Table 4:** Mean ADC values of the two scans per volunteer (selected single slice and region of interest); methods include protocol A and the corresponding average slice using protocol B with and without motion correction; ADC units 10^−5^ *mm* ^2^/*s*.

data-set	PA	AVG	LRA	NRA	LRA-b	AVG-b
v1-0503	113.90	113.67	108.35	109.27	107.53	112.64
v1-0515	127.66	105.36	105.51	108.91	105.45	104.18
v2-0521	119.22	111.43	99.45	109.45	102.19	111.32
v2-0528	129.53	105.74	102.61	105.95	99.52	105.50
v3-0524	115.24	100.60	93.38	95.67	92.39	98.51
v3-0528	104.82	97.54	89.86	92.35	88.96	96.31
v4-0611	129.42	101.63	92.54	94.47	91.65	100.98
v4-0614	129.89	99.30	95.23	92.88	93.74	97.72
v5-0731	104.68	78.72	73.65	76.21	71.58	76.70
v5-0809	105.81	77.57	76.57	76.65	75.25	75.77

**Fig 5:**
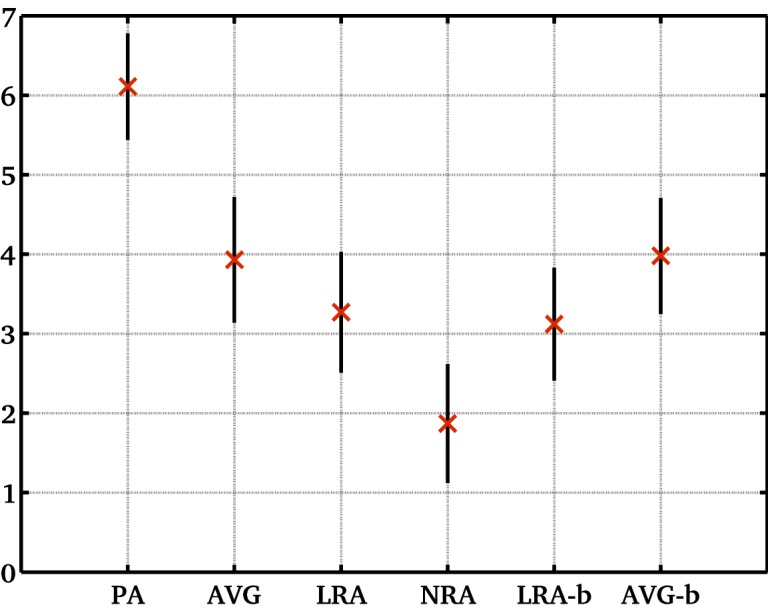
Mean ADC average reproducibility (%) within population for different methods. The within population average reproducibility (based on percentage change in mean ADC figures for different volunteers and methods as given in Table 3); methods include protocol A and the corresponding average slice using protocol B with and without motion correction; error bars show accuracy of each value).

**Fig 6:**
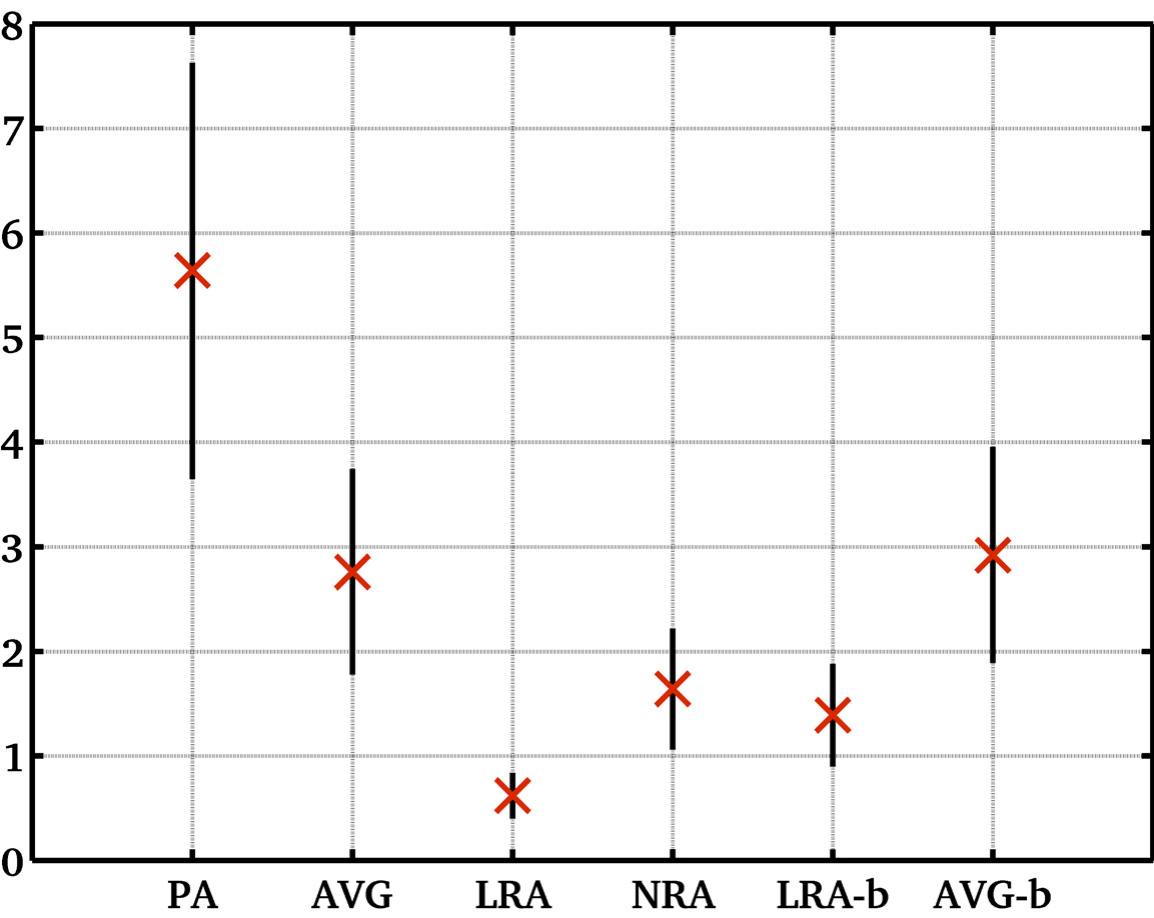
Mean ADC variability (%) within population for different methods. The within population average variability (based on percentage change in mean ADC figures for different volunteers and methods as given in Table 3); methods include protocol A and the corresponding average slice using protocol B with and without motion correction; error bars show accuracy of each value).

Figs 5–6 illustrate the accuracy of each measurement. We estimated the accuracy of average reproducibility figures using error propagation (Eq 11). This uses mean ADC values (Table 4), width of ADC histogram values (Table 1) and the number of entries in each ADC histogram *N*. Meanwhile, it is straightforward to compute the accuracy of each variability figure, as it is equivalent to the error on the standard deviation ($\sigma_{D}/\sqrt{2N_{s}}$ with *N*_*s*_ being the number of data-sets). Statistical errors of reproducibility (Fig 5) were small and similar (between 0.67 and 0.79) for different methods, while errors on variability (Fig 6) are significantly different for different methods (between 0.22 and 1.99).

## Discussion

Diffusion weighted MRI has become a standard diagnostic modality in a number of applications, such as acute stroke, where it is widely considered as a surrogate indicator of the relative size of the cellular component of the tissue. In practice a large range of factors, including: other tissue structural features such as fibrosis, necrosis or micro-cysts, the presence of local susceptibility variations, blood flow and methodological variations in acquisition technique can significantly affect estimated ADC values. This suggests that ADC is unlikely to provide a direct estimate of cell fraction, except in specific favourable circumstances. However, it is clear that changes in cell fraction can produce dramatic changes in ADC such as the restriction of ADC seen due to cytotoxic oedema in acute cerebral ischaemia. This has led to an interest in ADC as a biomarker of change in cellular fraction over time. In cancer applications a reliable biomarker of this type would be invaluable in the assessment of cellular proliferation or, more importantly in the assessment of cell kill in response to experimental therapies. Indeed, ADC has been widely used in clinical trials of radiotherapy cytotoxic and targeted novel agents.

In order to use ADC estimations to detect therapy induced changes it is essential that the technique has sufficient accuracy. This will depend on the underlying magnitude of the change, the sensitivity of the measurement technique and the sources and size of any measurement errors. It is clear from this and previous studies that, in the liver, physiological motion is the major source of error in ADC estimation. As percentage change in mean ADC (Δ*D*%) for individual fits in regions of good signal is often of the order of 5% (Table 3), the implication of these results is that motion is the dominant source of instability in ADC estimation.

The larger average reproducibility value corresponding to protocol A (6.11%) compared to NRA (1.87%) and LRA (3.27%) methods is a powerful illustration of the benefits of motion correction in these images. Further, the within population variability of the reproducibility metric was improved dramatically using LRA (0.62%) and NRA (1.64%) compared to protocol A (5.64%). This improvement is gained with no penalty in acquisition time since scan time would be comparable for protocol A and B if the same number of b-values, acquisition repeats and diffusion gradient directions were used. In quantitative studies the impact of this reduction in measurement error is profound. The accuracies obtained for the selected regions are on average consistent with our project target of 2–3% required for reliable detection of a 10% change in mean ADC.

Possible changes in the biological structures of tissues have been controlled by scanning each healthy volunteer twice (within two weeks), and using same regions of interest for each volunteer when applying motion correction and when computing the corresponding ADC reproducibility values. Hence the difference seen in the within population variability should be attributed to the use of different methods rather than being a reflection of underlying tissue heterogeneity. Using our approach, heterogeneous regions with broader ADC distributions (such as those seen in disease) would reduce sensitivity to the measurement of accuracy, but the apparent improvement due to better methodology of LRA over NRA would still be expected to be present.

We have demonstrated that LRA and its smoothed counterpart (LRA-b) show much better image quality and quantitative clustering of the parameter values from localised structures than NRA. Furthermore, the distribution of the fitted parameters matches that from NRA, while simple averaged (AVG) and its smoothed version (AVG-b) show a wider variability of parameters, probably reflecting measurement bias introduced by partial volume averaging.

LRA was comparable, and in some aspects superior, to the more sophisticated volume based non-rigid alignment. The significant amount of unwanted image smoothing in NRA images is caused by the need to estimate a large number of parameters using low SNR data and by the sub-sampling required to overcome odd-even slice misalignment and registration errors. However, measurement reproducibility was better with NRA than LRA, partly due to a reduced spread in measured ADC values caused by averaging. Blurring DW-MR slices could introduce artificial improvements in Δ*σ*_*D*_; further blurring of b-value slices corresponding to AVG (standard deviation 1.5 pixels Gaussian blurring) resulted in a significantly improved mean Δ*σ*_*D*_ to 19.10±0.65 compared to the AVG-b value of 15.88±0.51 (standard deviation 1 pixel) reported in Table 2. We would argue that given the choice of these methods, the LRA (non-smoothed) should be most suitable for assessment of ADC and particularly for the assessment of regional heterogeneity in ADC values.

One of the main disadvantages of a global registration technique is its computational cost. On a single computer, the typical processing time for one data-set can be several hours. The most suitable application for NRA currently therefore is offline usage. Another drawback of the NRA technique is that 3D global image registration implies re-sampling the registered images. This re-sampling involves interpolating the images, which is done twice in the case of NRA:the first for intra-image registration, the second for inter-image registration. In our study, the typical processing time for the NRA method,using an Intel Core i7 processor (8 CPUs, 2.7 GHz) and 16 GB of RAM, was 5 hours when applied to all data-sets corresponding to 3 b-values generating 120 (3 × 40) aligned slices. For the LRA method, using a similar machine (Intel Xeon processor X5660, 2.80 GHz with 24 GB of RAM), it took 46 seconds to generate 3 aligned slices (corresponding to 3 b-values). For both methods, source code could be slightly optimised and implementation could be parallelised to reduce execution time (an order of magnitude improvement is possible), but as the same kinds of gains are available to both methods, the order of magnitude ratio between these current figures would not be expected to change significantly. Using NRA, a few slices on the two edges of the volume could not be used for ADC analysis, as they could be corrupted by inevitable interpolation artefacts.

In clinical data with poor SNR, there may not be enough information in an image to support accurate determination of the large number of parameters needed for non-rigid alignment. Therefore, any practical application of registration methods needs an assessment of the required degree of complexity of the model. Simpler alignment methods may actually prove more suitable than generic ‘state-of-the-art’ techniques. The literature suggests several metrics for use in image alignment. However, the use of small image regions mitigates against the use of cost functions such as mutual information [38–41]. Getting good results in these tasks may not require a fundamental break-through in the state-of-the-art, but only pragmatic application of an appropriate technique.

As an alternative to the approach evaluated here, we may have considered motion correction by deconvolving the simple average image. To do this correctly, as movement patterns have been shown to have identifiable (linear) structure, we would require an estimate of the movement direction in order to construct an appropriate deconvolution kernel. Also it would seem an awkward approach to removing the effects of motion starting from a blurred image when the independent (sharp) images are available and only need to be moved into the correct positions. Deconvolution would therefore only be appropriate if it was impossible to obtain the separate images, but possible to estimate the motion in the region of interest. Note, however, that protocol A alone would not provide enough information to allow estimation of the required deconvolution kernel.

## Conclusions

Local-rigid alignment (LRA) is two orders of magnitude faster than non-rigid alignment (NRA), and so has the potential to be used in real-time in an ADC analysis pipeline just before the ADC measurement task. LRA works on a single slice basis while NRA works on the whole volume. However, LRA can also be used for larger scale (up to whole volume) motion correction tasks by setting up the required iterative process.

When it comes to heterogeneous tumours, it is recommended to use LRA rather than NRA to study single ADC measurements, region of interest ADC metrics (such as mean) or percentage change in ADC metrics. This is because LRA avoids blurring image data and maintains individual voxels corresponding to specific tissue during the alignment, while NRA is based on image interpolation which results in data blurring, and so changing the definition of final registered voxels (which may be difficult to interpret). Moreover, LRA results in a significantly greater reduction in the noise generated by motion than NRA.

Finally, it is recommended that data should be acquired using protocol B (off-scanner averaging) and not protocol A (on-scanner averaging), so that post-acquisition motion compensation can be applied to improve the measurement of percentage change in ADC metrics needed for clinical decisions on individual patients. It is part of our future research plan to investigate whether the methods discussed in this paper meet the specified reproducibility for detection of change in liver tumours.

## Supporting Information

S1 File The corresponding ISMRM 2015 abstract; reporting a short summary of our findings from the study presented in the current manuscript. (PDF)
