# Supplementary material for: The Accuracy of ADC Measurements in Liver Is Improved by a Tailored and Computationally Efficient Local-Rigid Registration Algorithm
Source: PLoS One. 2015 Jul 23;10(7):e0132554. doi: 10.1371/journal.pone.0132554 (PMC4512673; doi:10.1371/journal.pone.0132554)
Supplement: S1 File — (PDF) [file pone.0132554.s001.pdf]

1448

# AN EFFICIENT MOTION CORRECTION METHOD FOR IMPROVED ADC ESTIMATES IN THE ABDOMEN

Hossein Ragheb[1], Neil A. Thacker[1], Jean-Marie Guyader[2], Stefan Klein[2], and Alan Jackson[3]

*[1]Centre for Imaging Sciences, Faculty of Medical and Human Sciences, University of Manchester, Manchester, United Kingdom, [2]Biomedical Imaging Group Rotterdam, Departments of Medical Informatics and Radiology, Erasmus MC, Rotterdam, Netherlands, [3]The Wolfson Molecular Imaging Centre, Faculty of Medical and Human Sciences, University of Manchester, Manchester, United Kingdom*

**Target audience** – Those involved in the use of apparent diffusion coefficient (ADC) biomarkers from diffusion weighted MR (DW-MR) images in the abdomen, where respiratory motion causes movement during scans, for cancer drug trials and patient management.

**Purpose** – To provide an efficient method for retrospective motion correction suitable for use in a real-time pipeline prior to the measurement of voxel-by-voxel ADC values in a region of interest (ROI) in the abdomen. Also, to obtain more accurate ADC metrics in each ROI which maintain biological structure for purposes of later heterogeneity analysis. A novel, purpose designed local-rigid alignment (LRA) method is compared with a previously proposed, accurate but computationally expensive non-rigid alignment[1] (NRA). We evaluate both methods using metrics computed from regional ADC estimates on abdominal image slices of fasted healthy volunteers.

**Methods** – DW-MR images were acquired on a 1.5 T Siemens scanner. 5 fasted healthy volunteers were scanned twice within 2 weeks based on free breathing[2]. Two imaging protocols A and B and 3 b-values (100, 500 and 900 s/mm$^2$) were used. Protocol B provides 12 sets of 3D volumes: 4 repeated acquisitions with 3 separate diffusion gradient directions (per b-value). Protocol A provides one 3D volume per b-value for data averaged over the original 12 acquisitions. The LRA and NRA methods were applied to protocol B data to generate aligned b-value data. A single slice was selected from each of 10 data-sets for ADC measurement in manually defined ROIs (Fig. 1). These results were compared with those obtained using the corresponding protocol A data. NRA[1] performs an automatic global registration of 3D volumes using 3D non-rigid deformations. However, LRA aligns the data corresponding to the slice of interest. The method involves alignment of a single reference slice to all nearby repeat slices. The reference slice is selected from one of the lowest b-value images (best SNR) across the 3 gradient directions and 4 repeats. The ROI on which matching is performed covers the tissue on the left-hand side of the abdomen (including the liver) and above the level of spinal cord where motion occurs. This area contains various boundaries which provide the main information needed for matching. We set limits on the amount of motion present in the region, based on observations of respiratory motion (no more than 3 pixels in vertical and horizontal directions on each image slice and 2 slices along the z axis). Movement could be modelled on the basis of shifts of the origin. For each of the 3 gradient directions, 4 slices from the search neighbourhood are selected with best matching scores. These 12 slices were then averaged to obtain a motion compensated slice for a specific b-value. The process was repeated for other b-values using the same reference slice and constrained to find solutions consistent with the motion seen in the lowest b-value. Rather than using grey-level image patches directly, their gradients in the x and y directions were used. This reduces the dependency on absolute scaling of the data and is suitable for matching MRI data[3]. To measure ADC inside a ROI corresponding to a slice location, at each image pixel, signal values from the 3 b-value slices were fitted to an exponential curve (with a first order Rician correction). Mean ADC and standard deviation (SD) of ADC values were then computed within the ROIs, and a ΔSD measure was used to estimate the amount of random noise removed from data.

**Results** – Fig. 2 shows scatter plots of S0 signal against ADC obtained for an example data-set (Fig. 1) using 4 methods. In Table 1, we tabulate ΔSD between results obtained using simple averaging (AVG) and those obtained by applying LRA and NRA to protocol B data. Compared to NRA, LRA results in a larger modification to AVG, which is equivalent to SD of the noise removed. In Table 2, we tabulate the percentage change in mean ADC for each volunteer between the two scans. Here, NRA gives the best mean reproducibility, while LRA gives the lowest variability (SD) which is consistent with what we expect as a single scanner was used to scan fasted healthy volunteers. For comparison at equivalent levels of blurring, blurred versions of LRA and AVG (LRAb and AVGb) are included in Tables 1 and 2.

**Discussions** – Fig. 2 shows how the local regional correlations in parameters improve following registration. Investigation of the structures seen in these plots suggested that LRA performs best in maintaining the biological variation of data (see Fig. 1). NRA shows an overall distribution of the fitted parameters which matches the general variation seen for LRA, while AVG shows a wider variability of parameters. The results for mean ADC and SD (not shown here) obtained from protocol B data using AVG were consistent with those using protocol A (PA). We use regional ADC deviations to calculate the equivalent amount of random noise which would need to be added to data in order to obtain measurements as poor as AVG. The amounts of noise removed by registration and the difference between the alternative methods (based on mean ΔSD and its accuracy) are statistically significant. As the change in ADC for individual fits in regions of good signal is often of the order of 5%, the implication of these numbers is that motion is the dominant source of instability in ADC estimation. The figures for ADC reproducibility show a slight improvement in performance for NRA over LRA. However, NRA causes a significant amount of unwanted image smoothing[1]. Results for LRAb and AVGb suggest that blurring b-value slices could introduce artificial improvements in both mean ΔSD and mean reproducibility. LRA should therefore be more suitable for assessment of ADC heterogeneity. The figures obtained for the selected regions are on average consistent with the target of 3-4% required for reliable detection of a 10% change in ADC. Average reproducibility figures using PA are significantly larger than those using NRA and LRA. Hence, we can both tighten the ADC histogram using motion correction and achieve better reproducibility. Change in mean ADC is about two times larger for protocol A than for all the scenarios considered for protocol B.

**Conclusions** – LRA performs best in terms of recovering the genuine fine structures between the organs. This is more important if one requires ADC metrics from small ROIs (such as small tumours) in the proximity of these structures. LRA is sufficient to deal with the motion effects seen in this application and two orders of magnitude faster than NRA. It therefore has the potential to be used in real-time in an ADC analysis pipeline. Further, LRA results in a greater reduction in the noise generated by motion than NRA. It is recommended to use LRA rather than NRA to avoid interpolation. Finally, data should be acquired using protocol B and not protocol A, so that retrospective motion correction could be applied to improve the measurement of ADC change needed for clinical decisions on individual patients.

**References** – [1] J-M. Guyader *et al*. SPIE 9034, 2014. [2] D. Collins *et al*. IMI WP3 Task 2.3, 11/05/2012. [3] P.A. Bromiley *et al*. Frontiers in Zoology, 11(61), 2014.

**Acknowledgements** – Special thanks to David Collins and his colleagues at the Institute of Cancer Research who designed the imaging protocols and collected the data. This study received support from the Innovative Medicines Initiative Joint Undertaking (www.imi.europa.eu) under grant agreement number 115151, resources of which are composed of financial contribution from the European Union's Seventh Framework Programme (FP7/2007-2013) and EFPIA companies in kind contribution.

Fig 2. S0 versus ADC in the ROI (see Fig. 1).

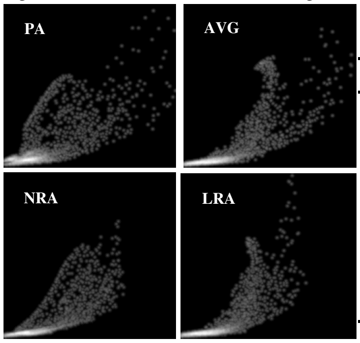


Table 1. ΔSD ($10^{-5}$mm$^2$/s): difference in the width of ADC distribution (SD) between that corresponding to AVG and those corresponding to alternative methods.

| data-set | LRA | NRA | LRAb | AVGb |
|---|---|---|---|---|
| v1-1 | 13.4 | 20.9 | 19.3 | 15.2 |
| v1-2 | 16.6 | 20.3 | 21.4 | 14.9 |
| v2-1 | 33.1 | -31.1 | 35.6 | 12.3 |
| v2-2 | 28.8 | -25.1 | 32.7 | 14.4 |
| v3-1 | 16.6 | 5.9 | 25.3 | 20.4 |
| v3-2 | 39.5 | 29.5 | 41.6 | 17.3 |
| v4-1 | 12.3 | 20.2 | 19.1 | 17.1 |
| v4-2 | 2.5 | 18.8 | 16.7 | 16.6 |
| v5-1 | -7.8 | 14.2 | 13.4 | 15.3 |
| v5-2 | -14.6 | 11.3 | 7.7 | 15.3 |
| mean±ac. | 14.0±4.2 | 8.5±4.9 | 23.3±2.5 | 15.9±0.5 |

Fig 1. Uncorrected and corrected b=100 s/mm$^2$ image slices and the ROI (v3-2).

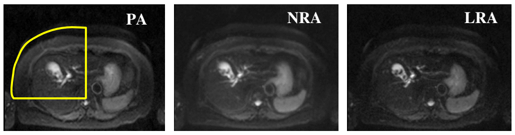


Table 2. Percentage change in mean ADC per volunteer per method.

| volunteer | PA | AVG | LRA | NRA | LRAb | AVGb |
|---|---|---|---|---|---|---|
| v1-1-2 | 11.4 | 7.6 | 2.6 | 0.3 | 1.9 | 7.8 |
| v2-1-2 | 8.3 | 5.2 | 3.1 | 3.2 | 2.6 | 5.4 |
| v3-1-2 | 9.5 | 3.1 | 3.8 | 3.5 | 3.8 | 2.2 |
| v4-1-2 | 0.4 | 2.3 | 2.9 | 1.7 | 2.2 | 3.3 |
| v5-1-2 | 1.1 | 1.5 | 3.9 | 0.6 | 5.0 | 1.2 |
| mean-SD | 6.1-5.6 | 3.9-2.8 | 3.3-0.6 | 1.9-1.6 | 3.1-1.4 | 4.0-2.9 |
